# Evaluating the value of entomotoxicology in forensic toxicology casework using the first minipig model

**DOI:** 10.1007/s11419-025-00728-1

**Published:** 2025-05-26

**Authors:** Olwen C. Groth, Anaëlle Pi, Andres E. Jensen, Frank Reckel, Jiri Hodecek, Abderrahmane Kori Yahia, Susan Rahaus, Martin H. Villet, Matthias Graw

**Affiliations:** 1 Institute of Forensic Medicine, Ludwig-Maximilians-Universität in Munich, Nussbaumstrasse 26, 80336 Munich, Germany; 2 University Côte d’Azur, 06108 Nice, France; 3 Ellegaard Göttingen Minipigs A/S, 4261 Dalmose, Denmark; 4 Bavarian State Criminal Police Office, Forensic Science Institute, Maillingerstrasse 15, 80636 Munich, Germany; 5 Swiss Human Institute of Forensic Taphonomy, University Centre of Legal Medicine, Chemin de La Vulliette 4, 1000 Lausanne, Switzerland; 6 Department of Pharmacy, Faculty of Pharmacy, University of Health Sciences, Algiers, Algeria; 7 Centre for Aviation and Space Medicine of the German Air Force, 51147 Cologne, Germany; 8 Department of Zoology and Entomology, Rhodes University, Makhanda, 6140 South Africa

**Keywords:** Diazepam, Forensic entomotoxicology, Göttingen Minipigs, *Lucilia sericata*, *Lucilia caesar*, Oxazepam

## Abstract

**Purpose:**

A principal objective of forensic entomotoxicology is to apply insect specimens for post-mortem toxicological analysis. Successful identification of drugs in necrophagous insects may depend on pharmacokinetic processes occurring in larvae. We thus applied a model system involving *Lucilia sericata* (Meigen, 1826) (Diptera, Calliphoridae) to investigate pharmacokinetics of diazepam in larvae in vitro, followed by a field experiment with Göttingen Minipigs.

**Methods:**

*Lucilia sericata* larvae were fed one of four diazepam concentrations at constant temperature, sampled regularly, and analysed for diazepam and metabolites by liquid chromatography tandem mass spectrometry (LC–MS/MS). Two Göttingen Minipigs of 60 kg each were euthanised one hour after oral administration of 25 mg/kg diazepam and placed outdoors. While available, samples of peripheral blood, cardiac blood, liver, and fly larvae were collected over 70 days. Extracts from porcine samples and larvae were analysed by LC–MS/MS. Some larvae were bred to adulthood and identified morphologically together with 718 larvae.

**Results:**

Oxazepam was a primary metabolite of diazepam in *L. sericata* larvae. The most prevalent fly species on minipig carcasses were *Lucilia caesar* (Linnaeus, 1758) (Diptera, Calliphoridae) and *Lucilia illustris* (Meigen, 1826) (Diptera, Calliphoridae). Diazepam and metabolites were detected in all larval samples, even weeks after porcine samples were unacquirable due to post-mortem decomposition. Ratios of oxazepam and nordazepam to diazepam concentrations in larvae were significantly higher than in associated porcine samples, confirming metabolism in larvae.

**Conclusion:**

These findings are relevant to forensic casework, as there is potential for misinterpreting that the deceased consumed oxazepam or nordazepam rather than diazepam. This caution may also apply to other drugs that can form through metabolism in larvae.

**Supplementary Information:**

The online version contains supplementary material available at 10.1007/s11419-025-00728-1.

## Introduction

Because of ethical constraints associated with the use of humans, several scientific disciplines employ animal models as proxies for experimental work that addresses issues pertinent to humans. While the “ideal” animal model remains a topic of debate [1–4], a close physiological and/or anatomical comparability to humans is generally a fundamental prerequisite. The selection of features of the animal model is primarily determined by the specific research question being investigated, which may encompass subjects that range from the exploration of therapeutic treatment strategies [5] to post-mortem forensic studies [6]. In forensics, the application of animal carcasses with physiology and anatomical size unlike those of humans can cause insect colonisation and decomposition patterns atypical of humans [7, 8]. For this reason, domestic pigs (*Sus scrofa* L.) were first suggested as an experimental model for forensic investigations more than 4 decades ago [9–11]. Even today, pig carcasses remain a preferred proxy for forensic entomologists [3, 12] and taphonomists [1] seeking data that can be extrapolated to human post-mortem cases with the greatest possible reliability [3, 10, 13]. Similarly, post-mortem toxicological and especially biomedical studies frequently use porcine models, because pigs share pharmacokinetic, pharmacodynamic, and other relevant characteristics with humans [14–16].

Forensic entomotoxicology (*i.e.* the study of necrophagous insects from a toxicological perspective [4, 17–19]) lies at the intersection of forensic entomology, forensic taphonomy, biomedicine, and post-mortem toxicology. Many authors applied intoxicated pig carcasses as human proxies for entomotoxicological research [4, 19]. In these studies, toxicants, including the psychoactive substances amitriptyline, citalopram, diazepam, morphine [20], and nicotine [21], and the inorganic poisons lead [22] and zinc phosphide [23] were administered to animals peri-mortem. Some of these investigations used carrion insects as proxy tissues for the detection of the target substances and their metabolites [20, 22, 23], but did not seek to elucidate how routine toxicological casework might benefit. At present, the practical value of using carrion insects for detecting toxicants in drug-related fatalities remains unclear. The lack of general agreement on whether the toxicological analysis of necrophagous insects from decomposed corpses can be beneficial in death investigations [24–27] underscores the need for further exploration on the topic.

In a study comparing toxicological results from human post-mortem samples with those obtained from larvae that fed on those corpses, it was argued that the analysis of larvae “is of almost no interest for practical forensic casework” because “drugs identified in maggots are always detectable in the cadaver too” [24]. However, as observed independently by Groth et al. and Peruch et al*.*, certain drugs and/or metabolites may be detected in larvae that are not identifiable in (most of) the numerous human specimens analysed [26, 27]. Traditional and/or suitable human samples for toxicological analysis may even be entirely absent in cases of advanced decomposition, animal scavenging, or relocation of the body. Here, insects that have fed on the cadaveric tissues may serve as alternative specimens to identify toxicants relevant to the fatality [27–29].

Even more factors argue both in favour of and against the use of necrophagous insects in toxicology casework. One consideration is that progressive metabolism, tropism, and excretion in the deceased and the larvae mean that no close correlation is to be expected between drug concentrations in larvae and dosages consumed by the deceased [4, 19, 24, 26], which poses a major limitation for toxicology casework. Conversely, some authors [30, 31] encountered less chromatographic interference and higher sensitivity from larval specimens than from the putrefied human samples they were collected from, thus offering a major benefit of using insects as additional or proxy matrices in cases of severe post-mortem decomposition. However, the successful detection of drugs in insects from a corpse is contingent upon several factors, including pharmacokinetic aspects in the insects. The ability of necrophagous insects to accumulate, metabolise, and eliminate drugs has been the subject of several past studies [32–34], but still requires further elucidation.

We, therefore, aimed to assess the application of necrophagous larvae in toxicological analyses at different stages of post-mortem decomposition using a porcine model as a human surrogate. The field experiment was preceded by an in vitro pilot study to first explore the pharmacokinetic characteristics of the target drug in necrophagous larvae under controlled laboratory conditions. *Lucilia sericata* (Meigen, 1826) (Diptera, Calliphoridae) was selected for the in vitro experiments due to its recommendation as an entomotoxicological model [4, 19], partly because of its significance in forensic entomology casework [35–37]. The benzodiazepine diazepam was selected as target drug, because of its relevance in forensic toxicology [38, 39]. Like other drugs in this class, diazepam is one of the most commonly prescribed psychoactive substances [40], which presents a social problem due to its high abuse potential and relevant role in drug-related deaths [41].

Among the porcine models used in biomedical research, miniature pigs (minipigs) are frequently a preferred subject for study. In addition to expressing some metabolic enzymes analogous to humans [42], minipigs are considerably smaller at sexual maturity than standard production pigs, which simplifies handling and reduces maintenance costs [42–44]. In the present study, sexually mature female minipigs were selected for their anatomical size, which is better representative of human bodies than that of juvenile males of the same species [45]. Minipig carcasses within the same size range as human corpses are expected to exhibit comparable patterns of decomposition and insect colonisation [3, 8, 10, 12]. The Göttingen Minipig, a crossbreed between the Minnesota minipig, the Vietnamese potbelly pig, and the German landrace pig, was developed specifically as a research model and is currently the most commonly used minipig breed in biomedical research [46]. Lignet et al*.* (2016) published an extensive investigation into the pharmacokinetics of diverse xenobiotics, including diazepam, in the Göttingen Minipig [47]. The authors report that the metabolic profile of diazepam in Göttingen Minipigs compares very well with that of humans [47].

We thus chose *L. sericata* and the Göttingen Minipig as model animals to investigate the potential of fly larvae for toxicological analysis, specifically in relation to detecting diazepam and its metabolites at different stages of post-mortem decomposition.

## Materials and methods

### In vitro metabolism study with *L. sericata* larvae

For comparability, the laboratory methods for diazepam fed to *L. sericata* larvae follow a previous published protocol [48], as follows.

#### Fly stock generation

For fly stock generation, *L. sericata* pupae were obtained from a commercial source (TerraristikShop.net, Düsseldorf, Germany). The eclosing adult flies were identified morphologically under a stereomicroscope (Leica MZ16, Leica Microsystems, Wetzlar, Germany), using a key for European and Mediterranean blowflies [49]. Adult flies were maintained at room temperature in rearing cages (35 × 21 × 21 cm) and were given sugar cubes and water (as soaked paper towels) ad libitum. Minced pork meat provided a protein source and substrate for oviposition.

Third-generation (F_2_) eggs of the same age were incubated together in a Memmert IPP 200 incubator (Schwabach, Germany) at 25(± 0.5) ℃ and 70(± 10)% relative humidity (RH). An artificial light:dark (L:D) cycle of 16:8 h was achieved using an LED light with automatic timer. From 12 h after oviposition, egg masses were monitored for freshly hatched larvae every hour to obtain first-instars of uniform age.

#### Rearing of larvae in the presence of diazepam

Batches of raw, minced pork meat were mixed with a diazepam (≥ 98%, Merck, Darmstadt, Germany) stock solution (1 g/L, *m/v*) in purified water (Milli-Q Millipore filter system, Bedford, MA, USA), which also contained hydrochloric acid (0.04 M HCl, Merck, Darmstadt, Germany) to facilitate diazepam solubility. A total volume of 4.8 mL, consisting of the required volume of diazepam stock solution and a 0.04 M solution of HCl in purified water, was added to each of four 240 g portions of minced meat to obtain the following concentrations: 0 µg/g, 2 µg/g, 10 µg/g, and 20 µg/g. These concentrations were based on diazepam concentrations reported in human specimens from real post-mortem cases [50–52]. Batches of meat were each homogenised in a food processor for 3 min, ensuring to avoid cross-contamination between batches. Batches were divided into three portions of 80 g each into plastic cups (Replicates i, ii, and iii).

Moist entomological brushes were used to transfer 100 freshly hatched *L. sericata* larvae to each of the 12 cups. For each of the four concentration treatments the three replicate cups with larvae were placed into a plastic container (18 × 11 × 12.5 cm), each of the four containers also holding a datalogger (FreeTec V2, Munich, Germany) for temperature and humidity measurement, and pet litter for pupation. The four plastic containers were each covered with a nylon net and incubated simultaneously (*t*_0_) at 25(± 0.5) ℃, 70(± 10)% RH, and 16:8 h L:D, as described above.

A beaker with minced meat, containing 2 µg/g diazepam and no larvae, was incubated concurrently as a control.

#### Sampling and storage of larvae and feeding medium

After incubation starting at t_0_, larvae were sampled and killed at the following intervals: four-hourly until 24 h (*t*_4_, *t*_8_, *t*_12_, *t*_16_, *t*_20_, and *t*_24_), six-hourly until 48 h (*t*_30_, *t*_36_, *t*_42_, and *t*_48_), 12-hourly until 96 h (*t*_60_, *t*_72_, *t*_84_, and *t*_96_), and 24 h later at 120 h (*t*_120_). Three larvae were collected for each sampling time and concentration treatment by taking one larva from each replicate container (i, ii, and iii). Larvae were killed by immersion in water at 90–100 ℃ for 60 s, followed by a washing step. After careful drying on paper towels, larvae from the same concentration treatment, sampled at the same time, were stored separately at − 20 ℃ until toxicological analysis.

Minced meat from the control beaker containing 2 µg/g diazepam and no larvae, and from the control beaker containing 2 µg/g diazepam with larvae, was sampled at the same intervals as larvae and stored at − 20 ℃ until toxicological analysis.

### Outdoor experiment with Göttingen Minipigs

A graphical abstract of the experimental procedure for the in situ experiments on the Göttingen Minipigs is provided in Fig. S1 in the Supporting Information.

#### Diazepam treatment of minipigs and first sampling of porcine specimens

Four 3-year-old female Göttingen Minipigs of 60(± 3) kg each were fasted for 24 h, after which two (Minipigs 2 and 3) received 25 mg/kg diazepam *per os*. Diazepam was fed in the form of crushed tablets (Diazepam DAK, 5 mg), mixed with 120 g of standard minipig diet pellets and 200 mL of commercially available apple juice. Two control minipigs (Minipigs 1 and 4) received the same amount of animal feed and apple juice without diazepam. All four minipigs were sacrificed after 1 h, which marks the estimated time for diazepam to reach maximum plasma concentrations in Göttingen Minipigs [47]. A licensed veterinary doctor sedated the animals with ketamine (10 mg/kg i.m.) and then euthanised them with an overdose of pentobarbital (140 mg/kg i.v.).

The first samples for toxicological analysis were taken directly after euthanasia (Day 0, D_0_) from each of the four minipigs. Peripheral blood from the jugular vein and cardiac blood (approximately 1 mL each) were taken by blind puncture, using disposable 10 mL syringes with 2 × 100 mm disposable needles. This method was chosen to minimise unusual bacterial and larval access resulting from excessive manipulation of the carcasses.

A small incision was made on the abdominal area of the minipigs to take liver samples of approximately 2 × 2 × 1 cm each. Incisions were sutured immediately after sampling to obstruct insects’ direct access to the internal organs and to minimise additional microbial contamination that may influence decomposition. Minipig carcasses were placed in individual containers, which were sealed and immediately transported to the experimental site according to European Union sanitary regulations (Regulation No. 1069/2009).

#### Carcass exposure

The study was conducted from 20 July (D_0_, day of diazepam treatment and euthanasia) to 27 September 2023 (Day 69, D_69_). Animal carcasses were exposed in a forest area with little human and wild animal activity starting from Day 1 (D_1_). The forest is situated south-west of Munich, Germany, and consists mostly of conifers, particularly spruce, followed by Weymouth pines and larches.

Each of the four minipig carcasses were placed in an individual cage (1235.5 × 83.5 × 97 cm, mesh size: 1 × 1 cm) that was supported on four feet, thus elevating the cages approximately 5 cm above the forest floor, according to the required regulations. Cages were spaced approximately 200 m apart in semi-shaded areas and at least 50 m away from the surrounding paths to minimise external influence and cross-contamination of dispersing larvae between carcasses [53]. Cages were secured with chains and locked to avoid access to wild animals and third parties. The two diazepam-treated minipigs were placed diagonally with respect to the two untreated minipigs (Fig. 2a). Four insect pitfall traps were positioned around each carcass, approximately 50 cm away, to collect wandering larvae.

### Sampling

Depending on availability, samples were taken from all four carcasses at each of 14 sampling intervals over a period of 69 days. Python version 3.1 was used to generate a randomised sampling order between the minipigs for each sampling day.

#### Sampling of biological specimens for toxicological analysis

Where available, fly larvae and porcine samples, including liver, cardiac blood, and peripheral blood from the jugular vein were taken at the following intervals post-mortem: daily during the first week (*D*_1_, *D*_2_, *D*_3_, *D*_4_, *D*_5_, *D*_6_, and *D*_7_), every second day until Day 13 (*D*_9_, *D*_11_, and *D*_13_), once weekly until Day 33 (*D*_20_, *D*_27_, and *D*_33_), and finally after one month (*D*_69_) (Fig. S1).

Fly larvae were collected in a pseudo-random manner, directly from the most colonised areas of the carcasses, irrespective of larval age. The specific area from which larvae were sampled until *D*_27_ depended on the availability of larvae at each sampling time point, and included the snout, the area around the abdominal incision, and areas below the legs (see Fig. 2). Spoons and blunt forceps were used for collection. No adult flies were collected during the sampling process. On *D*_33_ and *D*_69_, larvae were collected from within the minipig carcasses, because this stage of decomposition allowed larvae direct access to the internal organs, and from the insect traps around each carcass. All larvae destined for toxicological analysis were killed by immersion in near-boiling water, washed, dried on paper towels, and stored at – 20 ℃ until analysis, according to published recommendations [48].

Porcine specimens were taken as described in “Diazepam treatment of minipigs and first sampling of porcine specimens” for as long as blood and liver were available. As a result of post-mortem decomposition, some biological specimens were available for longer periods than others.

#### Sampling of fly larvae for species identification

Larvae were collected from each of the four minipig carcasses on D_5_, reared to adulthood on minced pork meat at 21 ℃ and 60–70% RH, killed in ethyl acetate, and identified morphologically using a published key for European and Mediterranean blowflies of forensic importance [54].

Approximately twenty randomly selected larvae from each of the four minipigs and each sampling day from *D*_7_–*D*_69_, inclusive, were killed by blanching in near-boiling water and stored in 70% ethanol until morphological identification. Larvae were identified under a Leica S9i stereoscope (Leica Microsystems, Wetzlar, Germany), using a 50 × magnification and a published key for European blowfly larvae of forensic importance [49]. Only third-instar larvae were identified to species level.

### Toxicological analysis

#### Sample extraction

(i) Extraction of drugs and metabolites from specimens in the in vitro experiment

An optimised method for extracting and subsequently detecting and quantifying benzodiazepines in larvae was applied. Larvae sampled from different concentration treatments and at the respective sampling time points were analysed separately, except for larvae sampled from 4 to 36 h (*i.e.* at time points *t*_4_, *t*_8_, *t*_12_, *t*_16_, *t*_20_, *t*_24_, *t*_30_, and *t*_36_), which were combined to provide approximately 100 mg of tissue per sample, and treated as representing the midpoint time of *t*_20_. Larvae from subsequent samplings were pooled according to their sampling times (*i.e.* *t*_42_, *t*_48_, *t*_60_, *t*_72_, *t*_84_, *t*_96_, and *t*_120_, respectively) and measured at least in triplicate per sampling time.

For each sample, larvae were weighed in a 2 mL disposable, reinforced Precellys^®^ vial (Bertin Technologies, Montigny-le-Brettonneux, France), to which 100 µL isotonic NaCl (≥ 99%, Roth, Karlsruhe, Germany) solution (0.9% *m/v*) in purified water (Milli-Q Millipore filter system) and five stainless steel beads (diameter: 2.8 mm) were added. Larvae were homogenised in a Precellys^®^ 24 tissue homogeniser at 4000 RPM for 90 s. After the addition of a deuterated internal standard (IS) mix, containing diazepam-*d*_5_, nordazepam-*d*_5_, oxazepam-*d*_5_, and temazepam-*d*_5_ (Ceriliant, Austin, TX, USA), the homogenate was loaded onto Oasis^®^ PRiME HLB 3cc cartridges (Waters GmbH, Eschborn, Germany) and slowly eluted with 2% formic acid (≥ 95%, Sigma–Aldrich Steinheim, Germany) in acetonitrile (99.9%, Sigma-Aldrich, Steinheim, Germany) under positive pressure, using the Waters Positive Pressure-96 Processor (Waters GmbH, Eschborn, Germany). The supernatant was evaporated to dryness at 37 ℃ under a stream of nitrogen and reconstituted in 150 µL of an aqueous ammonium formate solution (5 mM ammonium formate (≥ 97%, Honeywell Research Chemicals, Selzer, Germany) in purified water), containing 0.01% formic acid (≥ 95%, Sigma–Aldrich). Where necessary, extracts were passed through 0.45 µm VEREX_TM_ regenerated cellulose (RC) filters (Phenomenex^®^, Aschaffenburg, Germany) to remove undissolved particles before LC–MS/MS analysis.

Minced meat from control samples of 0 µg/g diazepam and 2 µg/g diazepam, one with and one without larvae, were treated as described for larvae.

(ii) Extraction of drugs and metabolites from larvae and porcine samples in the outdoor experiment

Where available, at least three samples per sampling event, minipig carcass, and matrix (*i.e.* larvae, porcine liver, cardiac blood, and peripheral blood) were extracted for analysis. Larvae were selected randomly for each sampling event and site, and purified as described for samples from the in vitro experiment. All porcine samples were purified by protein precipitation.

For protein precipitation 1.0 g of liver was first homogenised with 5 mL of isotonic NaCl solution for 5 min in an ULTRA-TURRAX^®^ (IKA, Staufen, Germany) and diluted 1:10 with liver homogenate (1.0 g liver + 5 mL isotonic NaCl solution) from untreated minipigs. After the addition of deuterated IS mix, 100 µL each of liver homogenate, peripheral blood, and cardiac blood were treated with 1 mL of acetonitrile, vortex mixed for 1 min, and centrifuged at 14,000 RPM for 5 min. The supernatant was evaporated to dryness at 37 ℃ under a stream of nitrogen and reconstituted in 150 µL of an aqueous ammonium formate solution (5 mM ammonium formate in purified water), containing 0.01% formic acid. Where necessary, extracts were passed through 0.45 µm VEREX_TM_ RC filters to remove undissolved particles before LC–MS/MS analysis.

#### Liquid chromatography–tandem mass spectrometry (LC–MS/MS)

Extracts were analysed by LC–MS/MS on an Agilent 6495 triple quadrupole (QQQ) system (Agilent Technologies, Waldbronn, Germany) for the detection and quantification of diazepam, nordazepam, oxazepam, and temazepam. Chromatographic separation was achieved on a Zorbax Eclipse Plus C_18_ column (Rapid Resolution HD, 2.1 × 50 mm, particle size: 1.8 µm) at 30 ℃. Five microliters of larval extract were injected. Due to small quantities of oxazepam and temazepam relative to diazepam in porcine samples, all extracts from porcine samples were injected twice: 5 µL for the detection and quantification of diazepam and nordazepam, followed by the injection of 10 µL of the same extract for the detection and quantification of oxazepam and temazepam. Mobile phases consisted of 5 mM ammonium formate in water with 0.01% formic acid (eluent A) and 0.01% formic acid in methanol (eluent B). A flow rate of 0.4 mL/min was used with the following gradient for eluent B: 0–1 min: 5–30%, 1–6 min: 30–60%, 6–8.5 min: 60–95%, 8.5–9.5 min: 95%, 9.5–9.51 min: 95–5%, and 9.51–12.5 min: 5% for re-equilibration.

Mass spectrometric analysis was performed in multiple reaction monitoring (MRM) mode with positive electrospray ionisation (AJS ESI +). Data were acquired and processed with the MassHunter software (Version 10.1). Identification criteria for diazepam, nordazepam, oxazepam, and temazepam included their retention times relative to those of the corresponding deuterated IS substances, two MRM transitions (diazepam *m/z* 192.9 and 153.9, nordazepam *m/z* 164.9 and 139.9, oxazepam *m/z* 268.9 and 240.9, and temazepam *m/z* 282.9 and 254.9), and the expected ion ratio (± 20%).

For quantification in larvae and porcine samples, the calibration curve for each analyte was created from at least eight calibration points (*R*^2^ ≥ 0.99) using extracts of drug-free larvae for larval samples and peripheral minipig blood from an untreated minipig for porcine samples that had been spiked with deuterated IS and undeuterated analytes in the corresponding concentrations. The calibration ranges for diazepam, nordazepam, oxazepam, and temazepam in both matrices were 100–2000 µg/L, 20–2000 µg/L, 50–1000 µg/L, and 50–500 µg/L, respectively. Final concentrations in larval and porcine samples were calculated from the ion ratio of the target ion to that of the corresponding deuterated IS and recalculated for the exact mass of larvae or minipig liver used for extraction. The limits of detection for diazepam, nordazepam, oxazepam, and temazepam are 13, 5, 11, and 6 µg/kg in larvae, and 10, 2, 10, and 5 µg/L in porcine blood.

Diazepam, nordazepam, oxazepam, and temazepam in minced meat from the in vitro experiments were analysed qualitatively.

#### Analysis of toxicological data from the in situ experiment

All samples from each day were averaged. Larvae were not always available from the same types of sites at each sampling event and due to post-mortem decomposition, the availability of porcine specimens also varied.

Because sample sizes of tissue matrices were unbalanced across the experiment, MANOVA-type analysis was not possible, and instead 95% confidence intervals (CI95) were calculated for each mean. If the CI95 for any two means did not overlap, the means were statistically significantly different at *α* = 0.05.

## Results

### In vitro metabolism study with *L. sericata* larvae

**Fig. 1:**
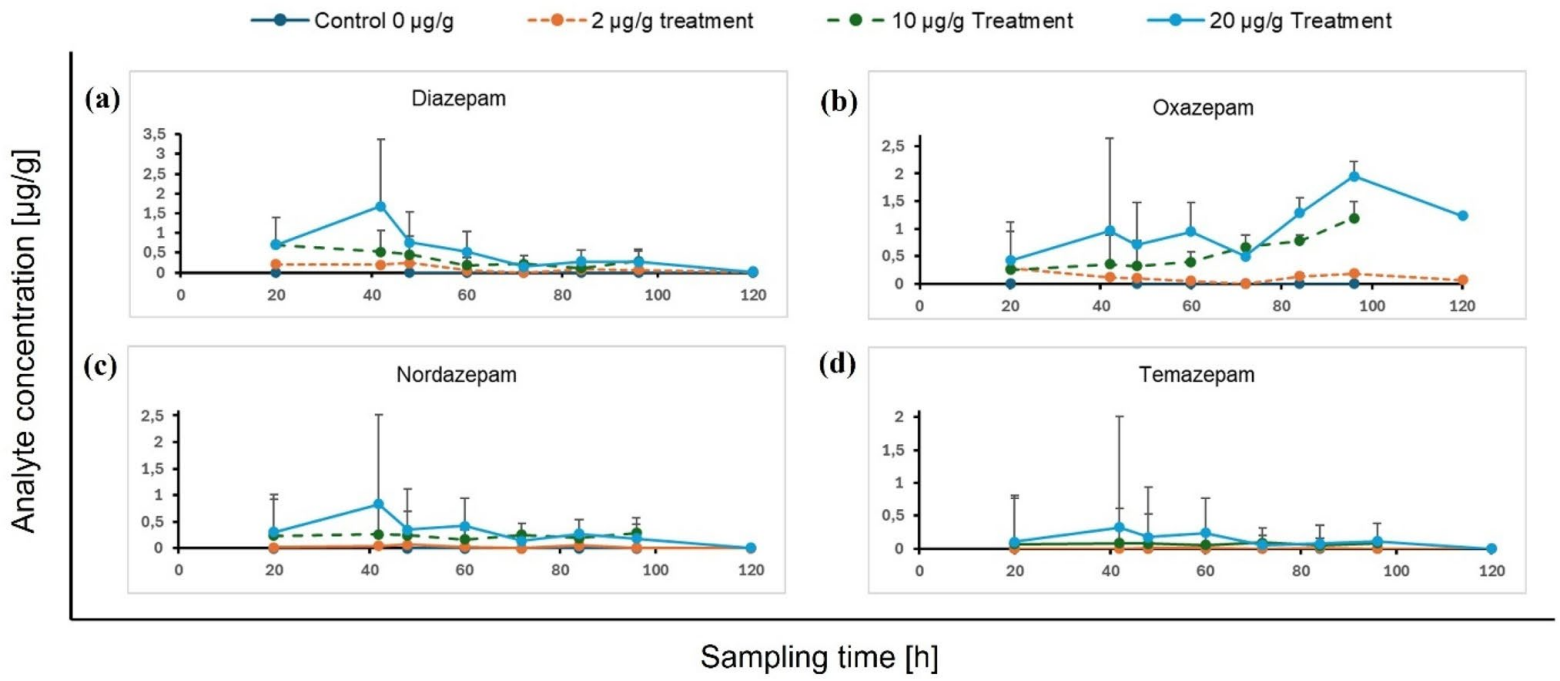
Stacked line charts of mean concentrations (95% confidence intervals) of **a** diazepam, **b** oxazepam, **c** nordazepam, and **d** temazepam detected in *L. sericata* larvae from different concentration treatments at specific sampling intervals. (20 h was taken as midpoint of the sampling times 4–36 h, representing concentrations of pooled samples that were taken at these sampling intervals; no larvae were available for analysis from the 10 µg/g treatment at sampling time 120 h)

Concentrations of diazepam and its metabolites nordazepam, oxazepam, and temazepam in larvae, sampled from the four different concentration treatments (*i.e.* 0 µg, 2 µg, 10 µg, and 20 µg diazepam per gram meat) as a function of sampling time are presented in Fig. 1.

Larvae from the 0 µg/g diazepam-treatment exhibited no detectable levels of diazepam or any of its metabolites. Minced meat control samples, containing 2 µg/g diazepam and no larvae yielded no detectable levels of nordazepam, oxazepam, and temazepam. Trace amounts of nordazepam, oxazepam, and temazepam were detectable in minced meat containing 2 µg/g diazepam that served as food source for larvae.

Concentrations of diazepam and metabolites in larvae fed with diazepam were initially proportional to diazepam concentrations in the food medium (Fig. 1a). Diazepam and nordazepam concentrations reached maximum levels in 42 h-old larvae, after which concentrations declined dramatically towards the post-feeding phase (Fig. 1a and c). In contrast, oxazepam concentrations continued to increase, only declining after 96 h of development (Fig. 1d). Temazepam concentrations remained relatively low throughout larval development (Fig. 1c). All substances except oxazepam were significantly less concentrated in post-feeding larvae than in younger larvae (Fig. 1).

### Outdoor experiment

#### Post-mortem decomposition

The average, minimum and maximum temperatures at the experimental site during the course of the outdoor experiment were 15.9, 1.6 (*D*_67_), and 31.7℃ (*D*_35_), respectively. The accumulated day degrees (ADD) by *D*_69_ was calculated to be 1148 ± 388 d ℃. The average rainfall over the course of the experiment was 3.9 mm, with peaks on *D*_37–40_ (average precipitation 30.9 mm), *D*_55_ (17.7 mm), and *D*_64_ (13 mm).

**Fig. 2:**
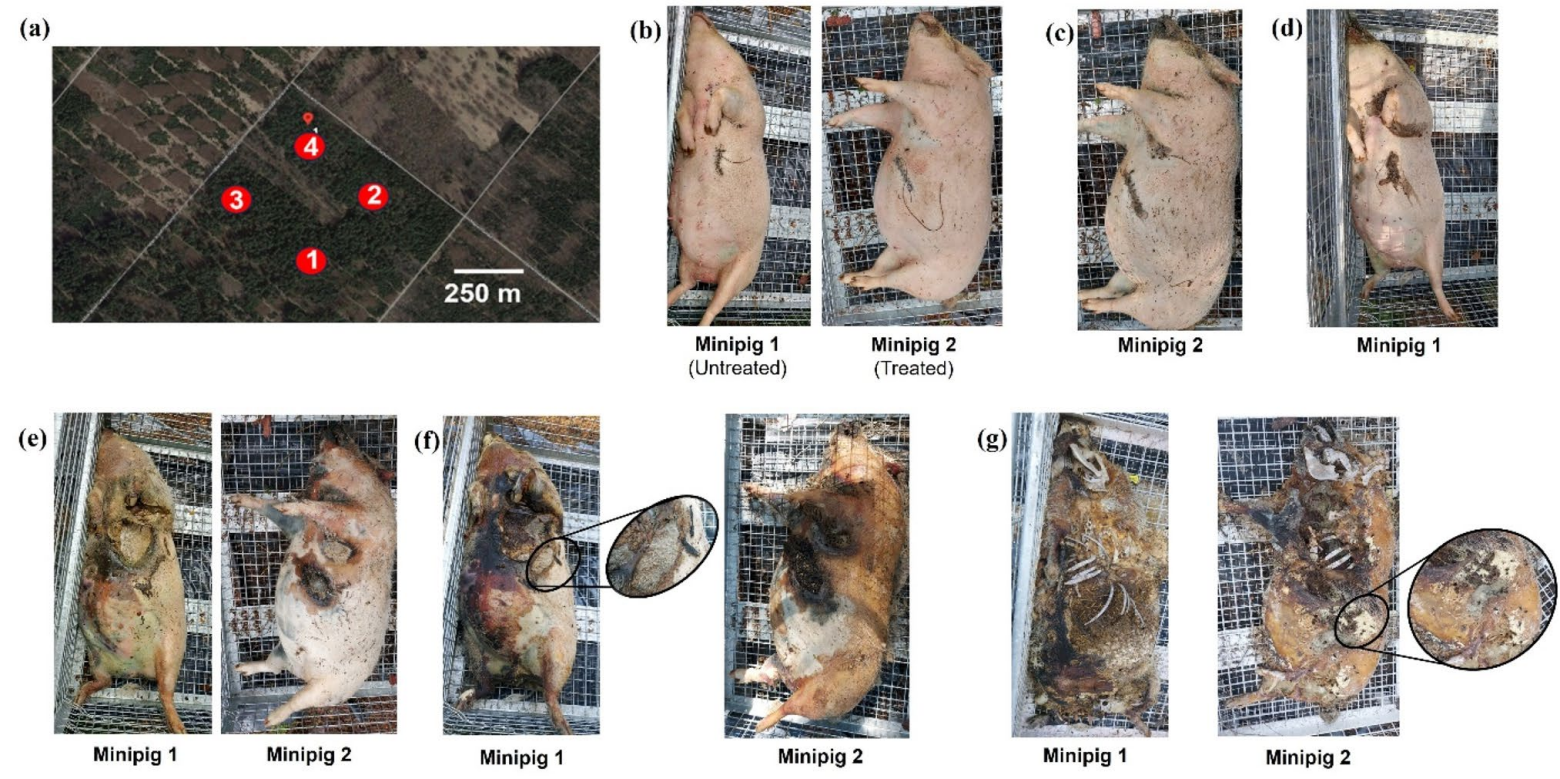
**a** Aerial view of the positions of treated (2, 3) and untreated (1, 4) minipig carcasses at the experimental site near Munich [58]. Progress of post-mortem decomposition and insect colonisation of the carcasses of a diazepam-treated (Minipig 2 in Fig. 2a) and an untreated (Minipig 1 in Fig. 2a) minipig on **b** *D*_5_, **c** *D*_9_, **d** *D*_11_, **e** *D*_20_, **f** *D*_27_ (showing actively feeding larvae), and **g** *D*_69_ (showing fly eggs)

The progress of decomposition and post-mortem insect colonisation for a diazepam-treated (Pos. 2 in Fig. 1) and an untreated minipig (Pos. 1 in Fig. 1) is demonstrated in Fig. 2, showing *D*_5_, *D*_9_, *D*_11_, *D*_20_, *D*_27_, and *D*_69_ after exposure on *D*_1_. All four carcasses showed a similar decomposition and insect colonisation pattern. Total body scores (TBS) of 25 were obtained for carcasses on D_69_, whether calculated according to the revised method for humans [55, 56], or using the method adapted for pig carcasses [57].

At least three replicates of peripheral blood of 100 µL each were available for toxicological analysis until *D*_9_, no usable peripheral blood samples were available on *D*_13_, and only one usable sample could be obtained on *D*_20_. At least three replicates of cardiac blood samples of 100 µL each were available until *D*_20_, and at least three replicates of liver samples were available until *D*_33_. Thus, no porcine samples were available for toxicological analysis after *D*_33_, whereas larvae were available for toxicological analysis continuously from *D*_4_ until termination of the experiment on *D*_69_.

#### Identification of fly species

Altogether, 87% of all enclosing images were identified as *Lucilia caesar* (Linnaeus, 1758) (Diptera, Calliphoridae), of which 63% were female. *Lucilia ampullacea* (Villeneuve, 1922) (Diptera, Calliphoridae) was the second most abundant species among survivors (11%), of which 57% were female. The remaining 2% were female, of which the species could not be determined.

Of the larvae collected and killed on *D*_7_, *D*_9_, *D*_11_, *D*_13_, *D*_20_, *D*_27_, *D*_33_, and *D*_69_, altogether 718 specimens were observed. Among these specimens, six species of Calliphoridae were identified, but larvae of *L. caesar* and *L. illustris* were indistinguishable [37]. Muscidae, Piophilidae, and Sarcophagidae together represented less than 5% of all fly larvae collected from *D*_7_–*D*_69_ (Table 1).

**Table 1:** Identification of fly larvae from minipig carcasses from all four minipig carcasses from *D*_7_, *D*_9_, *D*_11_, *D*_13_, *D*_20_, *D*_27_, *D*_33_, and *D*_69_, and post-feeding larvae from insect traps, collected on *D*_33_ and *D*_69_

Taxon	Number of individuals			Proportion	
	Family	Genus	Species	Of Calliphorids	Of all
**Calliphoridae**	**686**	–	–	**100%**	**95.5%**
*Lucilia*	–	101	–	14.7%	14.1%
* caesar/illustris*	–	–	100	14.6%	13.9%
* ampullacea*	–	–	1	0.14%	0.14%
*Calliphora*	–	88	–	12.8%	12.3%
* vomitoria*	–	–	79	11.5%	11.0%
* vicina*	–	–	9	1.31%	1.25%
*Protophormia*	–	11	–	1.60%	1.53%
* terraenovae*	–	–	11	1.60%	1.53%
Unknown*	–	486	–	70.9%	67.7%
**Muscidae**	**19**	–	–	–	**2.65%**
*Hydrotaea*	–	19	–	–	2.65%
* similis*	–	–	9	–	1.25%
sp.*	–	10	–	–	1.40%
**Piophilidae**	**5**	–	–	–	**0.70%**
**Sarcophagidae**	**8**	–	–	–	**1.11%**
* Sarcophaga*	–	8	–	–	1.11%

No Relevant difference was observed in the abundance of insect species between different minipig carcasses

*First and second-instar larvae were not identified to species level

Bold print indicates taxonomic families

### Detection of diazepam and metabolites in larvae and porcine specimens from minipig cadavers

**Fig. 3:**
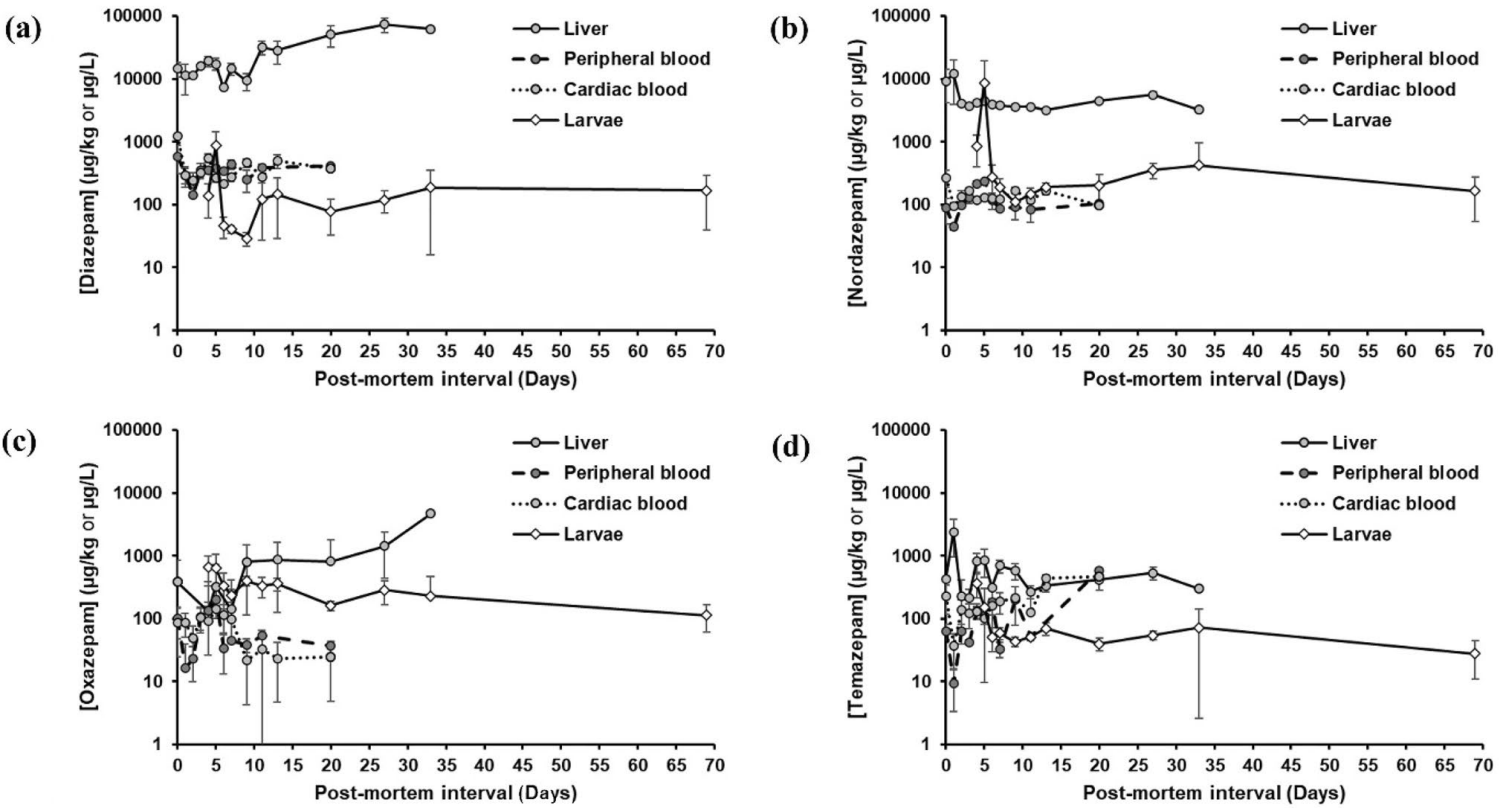
Mean concentrations (and 95% confidence intervals) of **a** diazepam, **b** nordazepam, **c** oxazepam, and **d** temazepam in porcine liver, peripheral blood, cardiac blood, and larvae as a function of sampling time. Points with 95% confidence intervals that do not overlap are statistically significantly different at *α* = 0.05

As illustrated in Fig. 3a, the concentrations of diazepam were significantly higher in liver samples compared to all other matrices. A comparison of liver specimens from differing sampling days revealed that those from *D*_11_–*D*_33_ yielded significantly more elevated diazepam concentrations than those originating from the first ten sampling days (*i.e.* *D*_0–9_) (Fig. 3a). Diazepam concentrations in peripheral blood were not significantly different from those in cardiac blood at any sampling time (Fig. 3a).

**Fig. 4:**
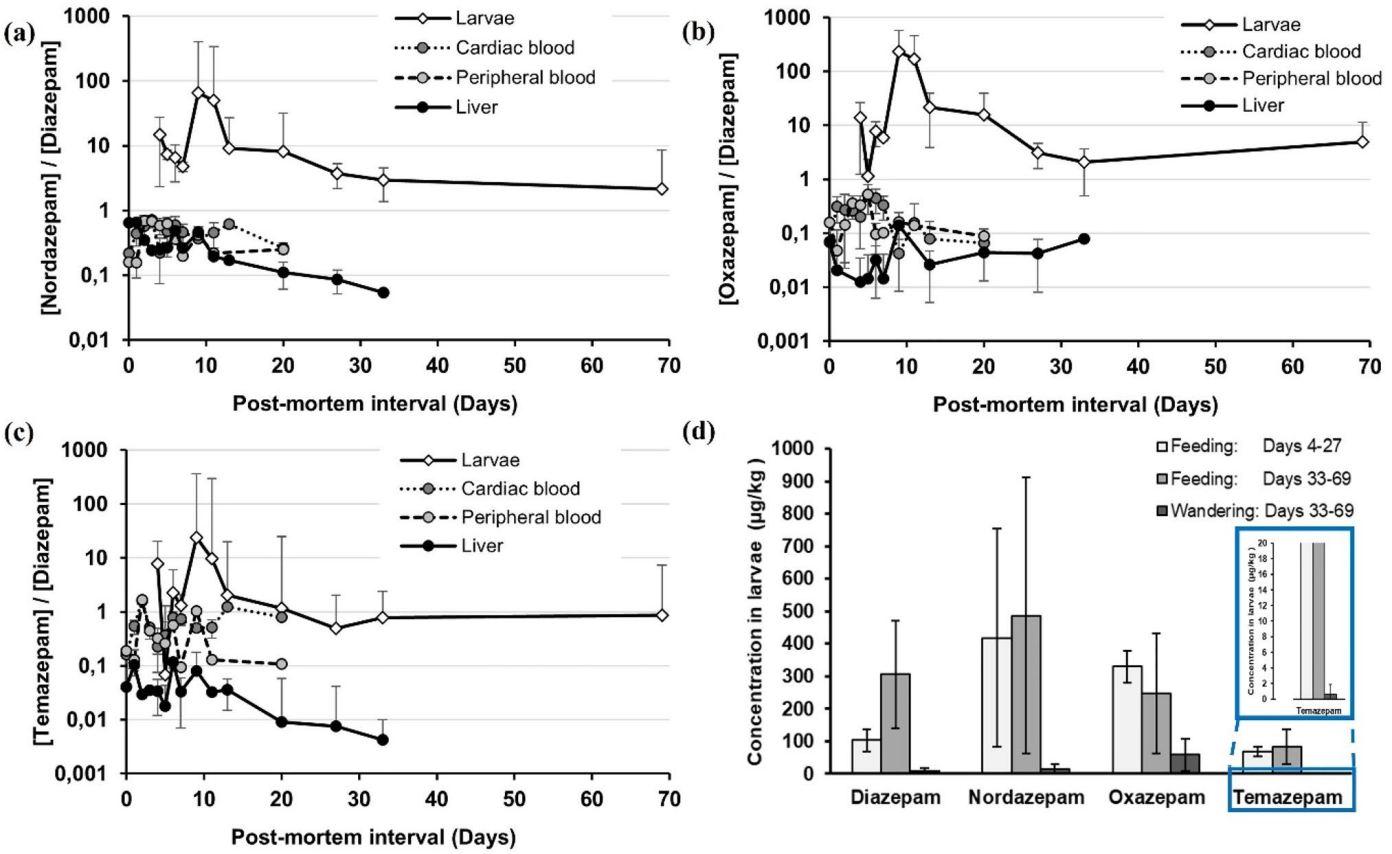
Mean concentration ratios (and 95% confidence intervals) of **a** nordazepam, **b** oxazepam, and **c** temazepam to diazepam in larvae compared to those in porcine specimens at different sampling times. **d** Mean concentrations (and 95% confidence intervals) of diazepam, nordazepam, oxazepam, and temazepam in feeding larvae from *D*_4–27_, compared to those in feeding and wandering larvae, respectively, sampled on *D*_33_ and *D*_69_. Points with 95% confidence intervals that do not overlap are statistically different at *α* = 0.05

Nordazepam concentrations were also significantly higher in liver specimens compared to the other matrices (Fig. 3b), except for *D*_5_, where concentrations were higher in larvae. Throughout the course of the experiment, markedly lower concentrations of oxazepam were detected in liver samples compared to those of diazepam (Fig. 3a and c; Fig. 4b). In fact, oxazepam was undetectable in 66% of all liver, 20% of all cardiac blood, and 7.6% of all peripheral blood samples analysed. A noteworthy observation was the increase in oxazepam concentrations in liver samples with an increase in post-mortem interval. Liver specimens from D_33_ yielded significantly higher oxazepam concentrations than samples collected at earlier post-mortem intervals (Fig. 3c).

Diazepam concentrations in larval samples were found to be lower than those detected in any of the three porcine matrices, except for *D*_5_ (Fig. 3a). For all larvae (*i.e.* over the entire sampling interval, whether actively feeding or wandering), the average nordazepam and oxazepam concentrations were higher than the corresponding diazepam concentrations (*i.e.* $\frac{[\text{nordazepam}]}{[\text{diazepam}] }>1 \text{and}$ $\frac{[\text{oxazepam}]}{[\text{diazepam}] }>1$) on all sampling days, a finding that was not observed in any of the porcine matrices (Fig. 4a and b). A similar observation was made when only feeding larvae, sampled between *D*_4_ and *D*_27_, were considered (Fig. 4d). However, larvae sampled later from the minipig carcasses (*i.e.* on *D*_33_ and *D*_69_) produced higher concentrations of diazepam relative to oxazepam, but diazepam concentrations remained low relative to nordazepam (Fig. 4d).

With the exception of oxazepam, all analyte concentrations in actively feeding larvae (*i.e.* larvae sampled directly from minipig carcasses on *D*_4–69_) were significantly higher than those in wandering larvae (*i.e.* larvae sampled from insect traps on *D*_33_ and *D*_69_) (Fig. 4d). Temazepam was not detectable in wandering larvae at all, and these exhibited only comparatively low concentrations of diazepam and nordazepam (Fig. 4d). However, no significant difference was found in average oxazepam concentrations between feeding larvae from *D*_33–69_ and wandering larvae sampled on the same days. A significantly higher oxazepam concentration was observed only between wandering larvae from *D*_33_ and *D*_69_ and feeding larvae from earlier post-mortem intervals (Fig. 4d).

## Discussion

### In vitro metabolism study of diazepam in *L. sericata* larvae

A detailed evaluation of diazepam pharmacokinetics during the early stages of larval development was not feasible due to the low body mass of young larvae.

**Fig. 5:**
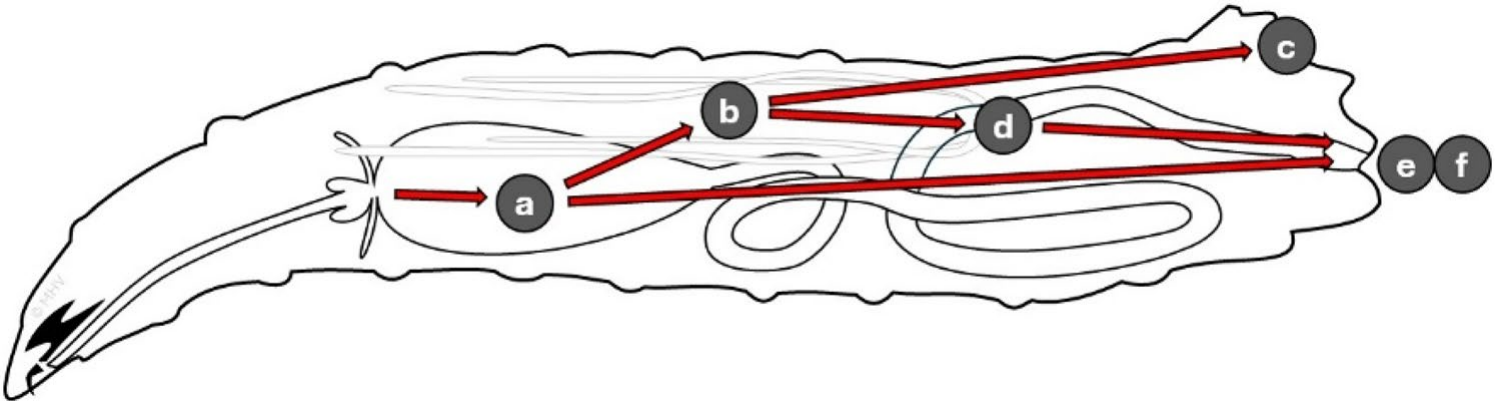
Diagram of a larva, facing left, showing its cuticular structures (exoskeleton and mouthparts: heavy black lines), gastrointestinal tract (light black lines), and processes of drug ingestion, metabolism, sequestration, excretion, and egestion (**a**–**f**). **a** Food bearing drugs is ingested and accumulated in the crop, where the drugs may be metabolised. **b** Some drugs and metabolites may be absorbed into the insect’s haemolymph and cytoplasm, where they may be metabolised by the larva. **c** Some drugs or their metabolites are sequestered in specialised tissues, *e.g.* in the cuticle, which is abandoned at ecdysis and eclosion. **d** Absorbed drugs and their metabolites may be excreted through the Malpighian tubules (indicated in grey) into the hind gut. **e** Unabsorbed drugs are directly egested along with faeces and other excreted xenobiotics. **f** Xenobiotics excreted during pupation may be sequestered in the hind gut lumen and eventually egested with meconium at eclosion

The time at which the larvae were sampled and the diazepam concentration in the medium both exerted a considerable influence on the concentrations of diazepam and metabolites detected in larvae. Diazepam and its metabolites were recovered well from larvae exposed to higher concentrations of the drug, in accord with past research [32] and process a in Fig. 5. Diazepam concentrations recovered from larvae Fig. 3a suggest that the ingestion and accumulation of diazepam (perhaps in the gut—Fig. 5, process a, or the haemolymph and cytoplasm—Fig. 5, process b) in *L. sericata* larvae exceeds its direct egestion (Fig. 5, process e) and metabolism (Fig. 5, processes b and a, respectively) between 42 and 48 h of development. Some drugs might even be subjected to metabolism by intestinal bacteria in the larval crop. This phase is followed by the cessation of feeding at the beginning of the post-feeding or wandering phase, which may (partially) account for the gradual decline in diazepam concentrations between 48 and 120 h (Fig. 3). Because wandering larvae defecate the last of their gut contents (and any unabsorbed diazepam that it contained—Fig. 5, process e), the presence of diazepam and metabolites in their bodies is evidence that they did in fact absorb the drugs (Fig. 5, process b).

The increase in oxazepam concentrations in larvae until 96 h, reaching levels above those of diazepam at any sampling time (Fig. 3d), demonstrates that the decline in diazepam concentrations over time is not solely a consequence of its egestion as wandering larvae empty their guts (Fig. 5, process e), but diazepam still undergoes active metabolism (Fig. 5, process b) until a few hours before pupation. As in humans, temazepam seems to be a minor metabolite in necrophagous larvae, as shown by the consistently low concentrations over time relative to the other analytes (Fig. 3c). Nordazepam followed a similar pattern to diazepam, albeit in a lower concentration range (Fig. 3b). Although substance concentrations in 120 h-old larvae were comparatively low (Fig. 3), which likely results from excretion and egestion (Fig. 5, processes d and e) or further metabolism (Fig. 5, process b), all analytes were still detectable until shortly before pupation. That the metabolites became less detectable means that they were not sequestered (Fig. 5, process c). Similar observations for several drugs were made in the necrophagous blowfly *Calliphora vicina* (Robineau-Desvoidy, 1830) (Diptera, Calliphoridae) [59, 60].

The absence of metabolites in diazepam-containing meat incubated without larvae and the presence of small amounts of nordazepam, oxazepam, and temazepam in diazepam-containing meat on which larvae were bred confirm that the detection of diazepam metabolites in larvae is a result of larvae metabolising the parent compound (Fig. 5, processes d and e), rather than an experimental artefact.

### In situ experiments with diazepam in Göttingen Minipigs

#### Fly species on minipig cadavers

Blowflies were by far the most abundant fly family on these minipig carcasses. The finding that *Lucilia caesar* was the most abundant species collected as larvae on *D*_5_ and bred to adulthood was anticipated, given that *L. caesar* typically frequents shady regions [61] such as the forest area that served as experimental site in the present study. Comparable distribution of insect fauna on pig carcasses [62] and human corpses [37] as ours (Table 1) were found by others in Central European forest habitats.

*Lucilia sericata*, the species used for the in vitro metabolism study of diazepam in necrophagous larvae, was not found on any of the minipig carcasses. This finding was not unexpected, because *L. sericata* is known to avoid forest areas and to prefer open and sunny habitats [61–63]. Nevertheless, *L. sericata* remains a good representative model to investigate larval drug metabolism in necrophages due to its nearly global distribution [4] and general prominence in post-mortem cases [36, 37].

#### Toxicological analysis of porcine specimens and larvae from minipig carcasses

(i) Diazepam and metabolites in porcine specimens

The effect of post-mortem redistribution of drugs is typically not as pronounced in blood sampled from peripheral veins [64]. For this reason, peripheral venous blood is generally preferred for quantitative analysis in post-mortem toxicology [64, 65]. However, in cases of severe post-mortem decomposition, blood may no longer be available from peripheral sources, so alternative matrices must be used [64]. Given the long duration of our field experiment, we thus chose a combination of peripheral blood from the jugular vein, cardiac blood, and liver as primary sources for quantitative analysis of diazepam and metabolites.

Peripheral and cardiac blood were sampled by blind puncture, to minimise the extent of manipulation to the carcasses, which may influence insect colonisation. In a study by Hargrove and McCutcheon [66], a comparison was made between drug concentrations (including those of diazepam) in blood taken by two methods. The authors established concentration ratios of approximately 1:1 between blood from clamped and unclamped veins, suggesting that results for diazepam of equivalent quality can be obtained from blood taken from unclamped veins. Consequently, it is postulated that the sampling technique employed in the present study would have no significant effect on the quantification of diazepam in porcine blood.

Diazepam does not exhibit significant post-mortem redistribution [52, 67], which is also reflected in the comparable concentrations between peripheral and cardiac blood throughout our experiment (Fig. 3a). However, a drastic increase in diazepam concentrations could be seen in the liver samples with increasing post-mortem interval, particularly during the second half of the experiment (Fig. 3a). The administration of a high oral dose of diazepam to the animals one hour prior to euthanasia resulted in elevated post-mortem concentrations of the drug in the stomach contents, leading to a concentration gradient relative to the surrounding tissues. Thus, the most probable explanation for the observed increase in diazepam levels in the liver tissues is post-mortem diffusion of gastric drug residue into the liver, leading to accumulation of the parent drug in hepatic tissues over time [68]. A similar observation was made in rats, which showed decreasing concentrations of diazepam in the gastro-intestinal tract, whereas these increased markedly in post-mortem liver specimens over 24 h [69]. Considering the anatomical discrepancies in hepatic and gastric anatomy, and the longer gastric emptying times [70], this effect may be more pronounced in Göttingen Minipigs.

Although oxazepam was undetectable in 66% of all liver samples, a significant increase in oxazepam concentrations was observed between the first 28 days of sampling (*i.e.* *D*_0–27_) and the last day when liver samples were available (*i.e.* *D*_33_) (Fig. 3c). In contrast to diazepam, this effect is unlikely to be the result of passive diffusion from gastric contents. During their investigation of the pharmacokinetics of diazepam in Göttingen Minipigs, Lignet et al*.* [47] identified temazepam (3-hydroxy-diazepam) and nordazepam (*N*-desmethyl-diazepam), together with three mono-oxidized glucuronide conjugates as the major metabolites in minipig plasma. Furthermore, glucuronidation was found to be a more pronounced metabolic pathway in vivo in minipigs [47]. It is, however, unclear, whether the authors detected oxazepam and its glucuronic acid conjugate in minipig plasma at all. We hypothesise that oxazepam is a minor metabolite of diazepam in Göttingen Minipigs, predominantly present in the glucuronated form. It is known that oxazepam conjugation and excretion occur rapidly in humans, at a rate that is nearly equivalent to its rate of generation after diazepam intake [71]. Considering the high glucuronidation activity exhibited by this species, the effect may be even more pronounced in Göttingen Minipigs, which would explain the low detection rate of oxazepam in our porcine samples. The significant increase in oxazepam concentrations in the liver samples from D_33_ may be due to microbial biotransformation from its conjugated form by intestinal bacteria. From a study of glucuronated benzodiazepine stability in dried blood spots, Wang et al*.* [72] concluded that temazepam glucuronide may not be subjected to the same transformation as oxazepam glucuronide, which may explain why liver temazepam concentrations did not increase with those of oxazepam (Fig. 3).

(ii) Diazepam and metabolites in larvae sampled from minipig carcasses and insect traps

The consistently higher oxazepam concentrations relative to the parent compound in larvae from the field experiment (Fig. 4b) are in accordance with those from our in vitro experiment with *L. sericata* (Fig. 1), thereby confirming that larvae of species other than *L. sericata* may also metabolise diazepam to form oxazepam.

Nordazepam concentrations in larvae from minipig carcasses were also elevated relative to diazepam (Fig. 4a), which was not observed in *L. sericata* in laboratory culture. However, unlike the in vitro experiments with only one species, a controlled analysis of diazepam pharmacokinetics was naturally not feasible in larvae from minipigs. Yet, the outdoor experiment is more representative of actual post-mortem cases. In a forensic setting, at any given time a combination of larvae from different species at differing stages of development would feed from varying areas of the body. Due to physiological variability, as well as ante- and post-mortem pharmacokinetics in the body, the larval feeding site would significantly influence the amount of diazepam (and metabolites) ingested by the larvae, which would, in turn, determine the extent of detectable concentrations in the insects. The same is true for the compilation of larval species in an outdoor setting, because different necrophagous insect species are believed to differ with regard to pharmacokinetic activity [4, 19, 73]. *Lucilia sericata* may thus exhibit a metabolic profile different from those species identified on the minipig carcasses, which would provide a rationale for the observed variation in nordazepam concentrations between the two experimental set-ups.

Despite potential differences in species, the two experiments had in common that wandering larvae yielded significantly lower concentrations of diazepam and its metabolites than actively feeding larvae. This phenomenon can be attributed to several processes in the larvae (Fig. 5) and to the fact that wandering larvae have ceased ingestion and completed egestion in preparation for pupariation [60, 74]. Oxazepam concentrations in wandering larvae from both experiments were found to be considerably higher relative to diazepam and nordazepam, whereas temazepam was nearly entirely undetectable in post-feeding larvae. In authentic forensic scenarios, such findings should be interpreted with caution, as the toxicologist may erroneously assume that the deceased had consumed the pharmacologically active metabolite (in this case, oxazepam or nordazepam), should these substances also be independently marketed.

Despite the reduced concentrations of diazepam and its metabolites in wandering larvae, these substances could still be identified in the insects nearly two months post-mortem, when standard specimens for toxicological analysis were no longer available. The high sensitivity of modern analytical techniques permits the successful detection of even very small drug quantities. Consequently, post-feeding larvae can be considered a promising option for toxicological analysis, *e.g.* if a corpse had been relocated.

The results presented in Fig. 3a demonstrate that there is no direct correlation between diazepam concentrations in larvae and those in minipig specimens, or the dose administered to the animals. At any given time, insects from the outdoor setting comprised several insect species at varying stages of development. Our in vitro experiment (Fig. 1) demonstrated that larval maturity exerts a significant influence on the concentrations of drugs and metabolites in larvae [4]. The phenomenon of unpredictable concentrations in larvae is further compounded by potential variations in pharmacokinetics of larvae from different species [4, 73]. Furthermore, as was also demonstrated in the in vitro experiment, drug concentrations in the food source also play a significant role in the quantitative results from larvae (Fig. 1). For the outdoor experiment, this would naturally depend on the larval feeding site, because diazepam and metabolite concentrations are not homogenously distributed throughout the carcass. As with authentic cases, larvae may only be available from restricted areas of the body, so selective sampling from specific areas may not always be possible. Furthermore, tropism may also play a role, as larvae may migrate over the carcass during the active feeding stage [4, 75].

Further discrepancies in larval concentrations include the higher diazepam and nordazepam concentrations relative to those of oxazepam in actively feeding larvae, sampled on *D*_33_ and *D*_69_, as compared to those in larvae from earlier post-mortem intervals (Fig. 4d). This effect may be a result of post-mortem decomposition of the minipig carcasses, causing larvae from *D*_33_ and *D*_69_ direct access to the viscera of the decomposing animals. Given the high degree of contamination of these tissues with diazepam from the stomach contents at the later stages of decomposition, it is evident that the levels of diazepam in these areas were considerably higher than in the peripheral regions of the carcass. Consequently, it can be inferred that the absorption of diazepam in the larvae was likely to be significantly higher than its conversion to oxazepam and the excretion of diazepam from the larval body.

The toxicological analysis of larvae from a corpse is thus unlikely to provide useful quantitative data that can be successfully extrapolated to the dose consumed by the deceased [24, 76, 77]. However, even primary tissue samples may become unreliable for quantitative extrapolation due to the effects of post-mortem physiological and environmental processes [4, 78]. Despite uncorrelated concentrations observed between primary porcine and proxy larval samples, diazepam, nordazepam, and oxazepam were still detectable in all larval samples, irrespective of the sampling time. This finding was particularly valuable during the latter stages of post-mortem decomposition, because no porcine samples were acquirable for toxicological analysis after *D*_33_, while larvae still offered a useful qualitative alternative. It is noteworthy that human cases have been documented where larvae were the sole specimens available for toxicological analysis [25, 26]. However, it is imperative to consider the metabolic processes in the larvae, particularly when the substance detected in the larval material is not only independently marketed, but also a metabolite of another substance.

### Study limitations

Site-to-site variability in drug concentrations is a known phenomenon in post-mortem toxicology [68]. To minimise the effects of drug diffusion from gastric contents on post-mortem analytical results in humans, it is recommended that liver samples be taken from deep within the right lobe [68]. In the current work, site-specific sampling of liver specimens was impractical, partially due to significant post-mortem bloating of the minipigs’ stomachs and the need to sustain as many liver samples as possible over the experimental period of 70 days. Liver specimens taken on the respective sampling days were thus acquired from different areas of the minipig liver to allow for many sampling events possible over the 70-day-long experimental period. Consequently, drug concentrations detected in the respective samples varied and are unlikely to be representative of the whole liver.

Similarly, xenobiotics in a carcass may affect decomposition and insect colonisation [79]. Rats killed with pentobarbital, the same method used in the present study, took twice as long to decompose as rats euthanised with carbon dioxide [80]. In addition, arthropod succession and development on rat carcasses were likely also influenced by the manner of death [80]. These effects of pentobarbital may also apply to our experiment with minipigs. However, in our experimental setting, barbiturate injection was the only acceptable technique for euthanasia of animals available to us.

## Conclusions

The present comparative toxicological study of diazepam and metabolites between samples obtained from decomposed minipig carcasses and larvae that fed off these remains demonstrates that necrophagous insect specimens can be successfully applied for qualitative toxicological analysis. The use of larvae as an alternative toxicological specimen proved particularly advantageous when standard primary sources were no longer available due to post-mortem decomposition. However, it is imperative to exercise caution when interpreting entomotoxicological results, particularly in instances where drug metabolites are also available on the market, because necrophagous larvae (and perhaps their bacteria) can metabolise parent compounds to produce such substances. This approach serves to prevent any potential misinterpretation of the drugs consumed by the deceased person.

This is the first entomotoxicological study to apply minipigs as human analogues. Due to anatomical, physiological, and pharmacokinetic similarities between Göttingen Minipigs and humans, these animals were a good proxy to prove the concept of using larvae for toxicological analysis. Nevertheless, such experiments should be confirmed in a human model, because of differences between the two models that may influence drug distribution and decomposition patterns [3].

Finally, the successful application of insect specimens for toxicological analysis and the consideration of drug effects on entomological PMI estimation require improved collaboration between forensic entomologists and toxicologists.

## Supplementary Information

Below is the link to the electronic supplementary material.

Supplementary file1 (DOCX 250 KB)
