# Supplementary material for: Evaluating the value of entomotoxicology in forensic toxicology casework using the first minipig model
Source: Forensic Toxicol. 2025 May 26;43(2):333–48. doi: 10.1007/s11419-025-00728-1 (PMC12241295; doi:10.1007/s11419-025-00728-1)
Supplement: Supplementary file 1 — Supplementary file1 (DOCX 250 KB) [file 11419_2025_728_MOESM1_ESM.docx]

**Supporting Information**

**Evaluating the value of entomotoxicology in forensic toxicology casework using the first minipig model**

Olwen C. Groth*^a^, Anaëlle Pi^a,b^, Andres E. Jensen^c^, Frank Reckel^d^, Jiri Hodecek^e^, Abderrahmane Kori Yahia^a,f^, Susan Rahaus^a,g^, Martin H. Villet^h^, Matthias Graw^a^

^a^Institute of Forensic Medicine, Ludwig-Maximilians-Universität in Munich, Nussbaumstrasse 26, D-80336 Munich, Germany

^b^University Côte d'Azur, 06108 Nice, France

^c^Ellegaard Göttingen Minipigs A/S, 4261 Dalmose, Denmark

^d^Bavarian State Criminal Police Office, Forensic Science Institute, Maillingerstrasse 15, D-80636 Munich, Germany

^e^Swiss Human Institute of Forensic Taphonomy, University Centre of Legal Medicine, Chemin de la Vulliette 4, CH-1000 Lausanne, Switzerland

^f^Department of Pharmacy, Faculty of Pharmacy, University of Health Sciences, Algiers, Algeria

^g^Centre for Aviation and Space Medicine of the German Air Force, D-51147 Cologne, Germany

^h^Department of Zoology & Entomology, Rhodes University, Makhanda, 6140 South Africa

ORCID 0000-0002-4335-5667; [martin.villet@gmail.com](mailto:martin.villet@gmail.com)

*Corresponding author

ORCID 0000-0003-4672-041X

*E-mail address:* [olwen.groth@med.uni-muenchen.de](mailto:olwen.groth@med.uni-muenchen.de)

*Telephone number:* (+49) 89 2180 73221


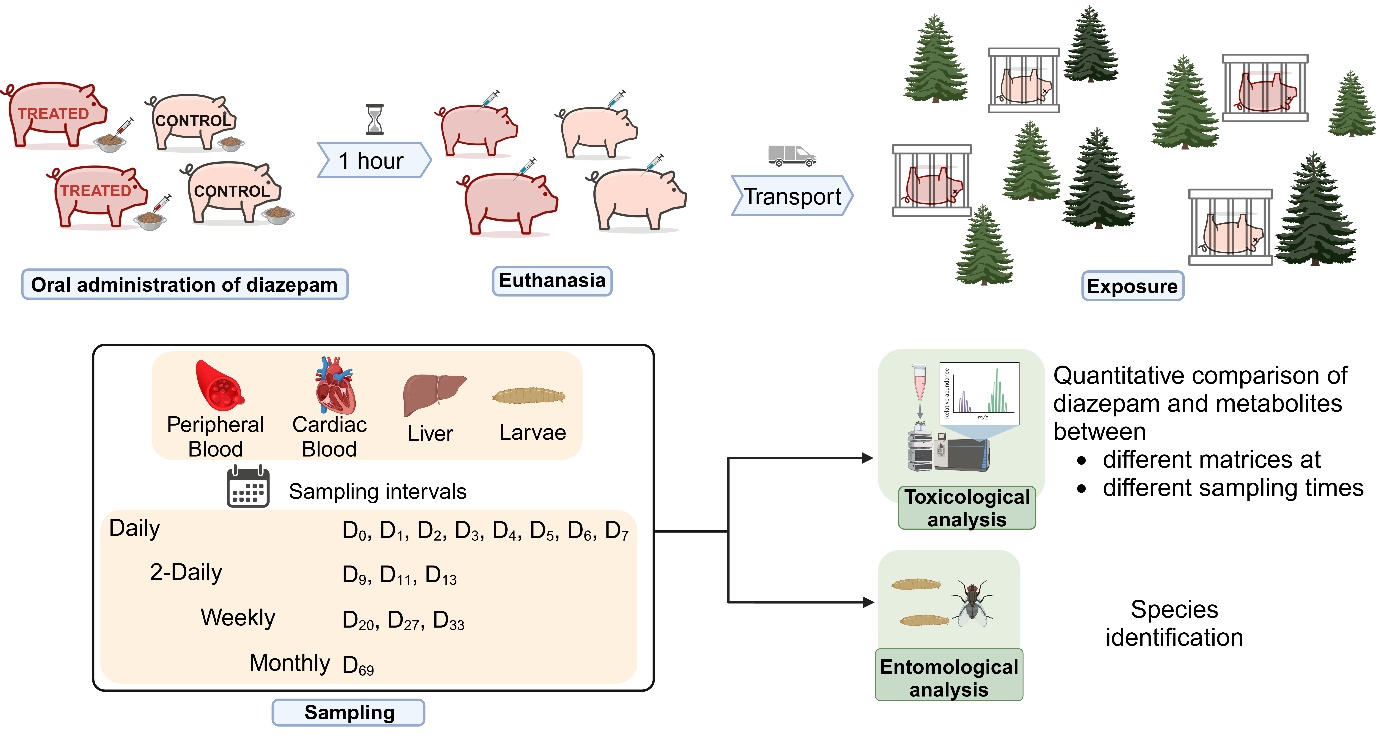


**Fig. S1** Design of the *in situ* experiment, showing treatment of two Göttingen Minipigs with diazepam and simultaneous euthanasia of the treated and two untreated minipigs, followed by transport and exposure of the four carcasses at the experimental site. Sampling of porcine samples and larvae was performed regularly, followed by toxicological and entomological analyses [1]

**References**

1. Pi A (2025) Created in BioRender. https://BioRender.com/z05s116
